# EEG/ERP evidence of possible hyperexcitability in older adults with elevated beta-amyloid

**DOI:** 10.1186/s40035-022-00282-5

**Published:** 2022-02-09

**Authors:** Hannes Devos, Kathleen Gustafson, Ke Liao, Pedram Ahmadnezhad, Bradley Estes, Laura E. Martin, Jonathan D. Mahnken, William M. Brooks, Jeffrey M. Burns

**Affiliations:** 1 Department of Physical Therapy, Rehabilitation Science, and Athletic Training, University of Kansas Medical Center, Kansas City, KS 66160 USA; 2 Department of Neurology, University of Kansas Medical Center, Kansas City, KS 66160 USA; 3 Hoglund Biomedical Imaging Center, University of Kansas Medical Center, Kansas City, KS 66160 USA; 4 Department of Population Health, University of Kansas Medical Center, Kansas City, KS 66160 USA; 5 Department of Biostatistics and Data Science, University of Kansas Medical Center, Kansas City, KS 66160 USA; 6 University of Kansas Alzheimer’s Disease Center, University of Kansas Medical Center, Kansas City, KS 66160 USA

**Keywords:** Event-related potentials, Electro-encephalography, Working memory, Older adults, Preclinical, Beta-amyloid

## Abstract

**Background:**

Although growing evidence links beta-amyloid (Aβ) and neuronal hyperexcitability in preclinical mouse models of Alzheimer’s disease (AD), a similar association in humans is yet to be established. The first aim of the study was to determine the association between elevated Aβ (Aβ+) and cognitive processes measured by the P3 event-related potential (ERP) in cognitively normal (CN) older adults. The second aim was to compare the event-related power between CNAβ+ and CNAβ−.

**Methods:**

Seventeen CNAβ+ participants (age: 73 ± 5, 11 females, Montreal Cognitive Assessment [MoCA] score 26 ± 2) and 17 CNAβ- participants group-matched for age, sex, and MOCA completed a working memory task (*n*-back with *n* = 0, 1, 2) test while wearing a 256-channel electro-encephalography net. P3 peak amplitude and latency of the target, nontarget and task difference effect (nontarget−target), and event-related power in the delta, theta, alpha, and beta bands, extracted from Fz, Cz, and Pz, were compared between groups using linear mixed models. P3 amplitude of the task difference effect at Fz and event-related power in the delta band were considered main outcomes. Correlations of mean Aβ standard uptake value ratios (SUVR) using positron emission tomography with P3 amplitude and latency of the task difference effect were analyzed using Pearson Correlation Coefficient *r*.

**Results:**

The P3 peak amplitude of the task difference effect at Fz was lower in the CNAβ+ group (*P* = 0.048). Similarly, power was lower in the delta band for nontargets at Fz in the CNAβ+ participants (*P* = 0.04). The CNAβ+ participants also demonstrated higher theta and alpha power in channels at Cz and Pz, but no changes in P3 ERP. Strong correlations were found between the mean Aβ SUVR and the latency of the 1-back (*r* =  − 0.69; *P* = 0.003) and 2-back (*r* =  − 0.69; *P* = 0.004) of the task difference effect at channel Fz in the CNAβ+ group.

**Conclusions:**

Our data suggest that the elevated amyloid in cognitively normal older adults is associated with neuronal hyperexcitability. The decreased P3 task difference likely reflects early impairments in working memory processes. Further research is warranted to determine the validity of ERP in predicting clinical, neurobiological, and functional manifestations of AD.

**Supplementary Information:**

The online version contains supplementary material available at 10.1186/s40035-022-00282-5.

## Introduction

Alzheimer’s disease (AD) is increasingly viewed as a disconnection syndrome leading to reduced communication between brain areas [1, 2]. Emerging evidence shows that the reduced neurotransmission is caused by the disturbance of the synaptic excitation/inhibition balance in the brain [1, 2]. Even in the preclinical phase when no cognitive impairments are apparent [3], beta-amyloid (Aβ) oligomers and Aβ plaques show associations with this excitation/inhibition imbalance and altered activity of local neuronal circuits and large-scale networks [4]. Preclinical mouse models of AD support the notion that this imbalance causes hyperactivity in hippocampal and cortical neurons and reductions of slow-wave oscillations, even before the appearance of Aβ plaques [4]. Such hyperactivity shifts the normal excitation/inhibition balance towards neuronal hyperexcitability, mediated through both increased excitation of synaptic glutamatergic tone and decreased GABAergic inhibition [4]. This relative neuronal hyperexcitability in turn leads to excitotoxicity [5] and amplification of synaptic release of Aβ [6], ultimately leading to further neurodegeneration and neuronal silencing mediated by concomitant tau accumulation [7]. Previous studies have explained this hyperexcitability as a physiological compensation for the increased Aβ burden in preclinical AD [8–11], wherein the accumulation of Aβ deposits results in neural recruitment, up until a certain threshold when the compensatory mechanisms fail. The hyperexcitability is then followed by hypoexcitability due to functional neuronal silencing in clinically diagnosed AD [7].

Electro-encephalography (EEG) offers insights into the postsynaptic activity of pyramidal cells and may therefore be useful for evaluating the impact of Aβ deposits on neuronal excitability in older adults across the spectrum of AD [12]. A systematic review of published studies has shown consistent evidence of hypoexcitability in AD, expressed as reduced power in the high-frequency bands, and lower amplitude and larger latency of event-related potentials (ERP) [12]. The associations between Aβ and neuronal excitability in mild cognitive impairment (MCI) and preclinical AD are less clear. One resting-state EEG study including older adults with subjective memory impairments has found a non-linear relationship between Aβ and delta power, in those individuals who showed signs of neurodegeneration, but not in those with normal-appearing brain [11]. Another study has shown that older adults with increased Aβ load and subjective cognitive impairments exhibit greater connectivity in the alpha band and reduced connectivity in the beta band [13]. Studies evaluating excitability under cognitive load in the cognitively normal (CN) older adults are even more limited. In a previous study, the event-related spectral power in the alpha and beta bands extracted while doing a working memory task (2-back) was higher in older adults with unknown Aβ status who showed deterioration in an 18-month follow-up assessment compared to CN participants who remained stable [14]. Although previous work suggests neuronal hyperexcitability in preclinical AD, possible changes in the event-related power in cognitively normal, amyloid elevated (CNAβ+) older adults are yet to be established.

Previous research suggests that the changes in spectral frequency due to increased amyloid burden reflect initial compensatory processes to maintain normal cognitive function [8, 9, 11]. However, it is unclear how neuronal hyperexcitability affects the efficiency of cognitive processing. ERPs offer unique insights into the neural processes of working memory under cognitive load. The P3 (or P300) is a positive ERP that appears at around 300 ms after stimulus onset. The amplitude of P3 is generally considered as a measure of resource allocation, particularly during working memory tests [15]. Higher cognitive demands result in decreased P3 amplitudes and longer latencies [16]. The attenuated P3 amplitude with increased cognitive demand is explained by the reallocation of resources away from the stimulus discrimination task towards processes that are more responsible for the higher demands posed on working memory, such as information storage and updating [17]. The P3 component can be isolated by discriminating the frequent nontarget from the infrequent target. This task difference effect reflects frontal lobe activity that is sensitive to the attentional demands induced by the task [18]. Larger task difference effects reflect more efficient discrimination ability in the stimulus evaluation process.

The main aim of this study was to compare the physiological response during working memory tasks of incremental cognitive demand between CN older adults with and without increased Aβ load. We hypothesized that CNAβ+ participants would show decreased P3 amplitude of the task difference effect compared to CN non-elevated (CNAβ−) participants. In a previous study [19], we established the reliability of the P3 ERP of the task difference effect at Fz in older adults with and without cognitive impairments. Therefore, we predesignated the P3 amplitude of the task difference effect at channel Fz as the main outcome variable, but also calculated the amplitude and latency of nontarget and target responses at channel Fz and additional midline channels Cz and Pz. The second aim of this study was to compare the event-related power between CNAβ+ and CNAβ− participants. Due to the early breakdown of slow-wave frequency bands shown in animals with preclinical AD [4], we expect lower event-related power in the delta band in CNAβ+. Since hypoexcitability in clinically diagnosed AD manifests as decreased power in the higher frequency bands, we expect increased power in the alpha and beta bands to reflect hyperexcitability in CNAβ+. Finally, we explored the association between Aβ uptake and P3 peak amplitude and latency of the task difference effect.

## Materials and methods

### Participants

All participants were recruited from the University of Kansas Alzheimer’s Disease Center between May 30, 2018 and July 20, 2020. Participants were excluded if they (1) were currently taking steroids, benzodiazepines, or neuroleptics; (2) had a history of any substance abuse; (3) had a history of a neurological disorder; or (4) had any contra-indications to positron emission tomography (PET) or EEG. The inclusion criteria were (1) age of 65 years or older; (2) understanding all instructions in English; (3) having given informed consent; and (4) a previously administered amyloid PET scan of the brain. The cerebral amyloid burden was assessed using PET images, obtained on a GE Discovery ST-16 PET/CT scanner after administration of intravenous Florbetapir ^18^F-AV45 (370 MBq) following a previously published protocol [20]. To determine the Aβ status, three experienced raters interpreted all PET images independently and without reference to any clinical information, as previously described [21]. The final status was determined by the majority of raters as Aβ− versus Aβ+, using a process that combined both visual and quantitative information [22, 23]. The median (Q1–Q3) time between the PET scan and EEG assessment was 1111 (794–1675) days.

### Procedure

#### Demographic and clinical information

We recorded information of age, sex, education, race, and ethnicity of participants. The participants also completed the Montreal Cognitive Assessment (MoCA) as a general screen of cognitive functions, which was carried out by a member of the research team who was blinded to the group allocation [24]. Normal cognition was confirmed after a clinical assessment performed at the time around PET scan at the University of Kansas Alzheimer’s Disease Center, which included the Clinical Dementia Rating [25] and Uniform Data Set Neuropsychological Battery [26]. All participants reported to be right-hand dominant.

#### N-back test

**Fig. 1:**
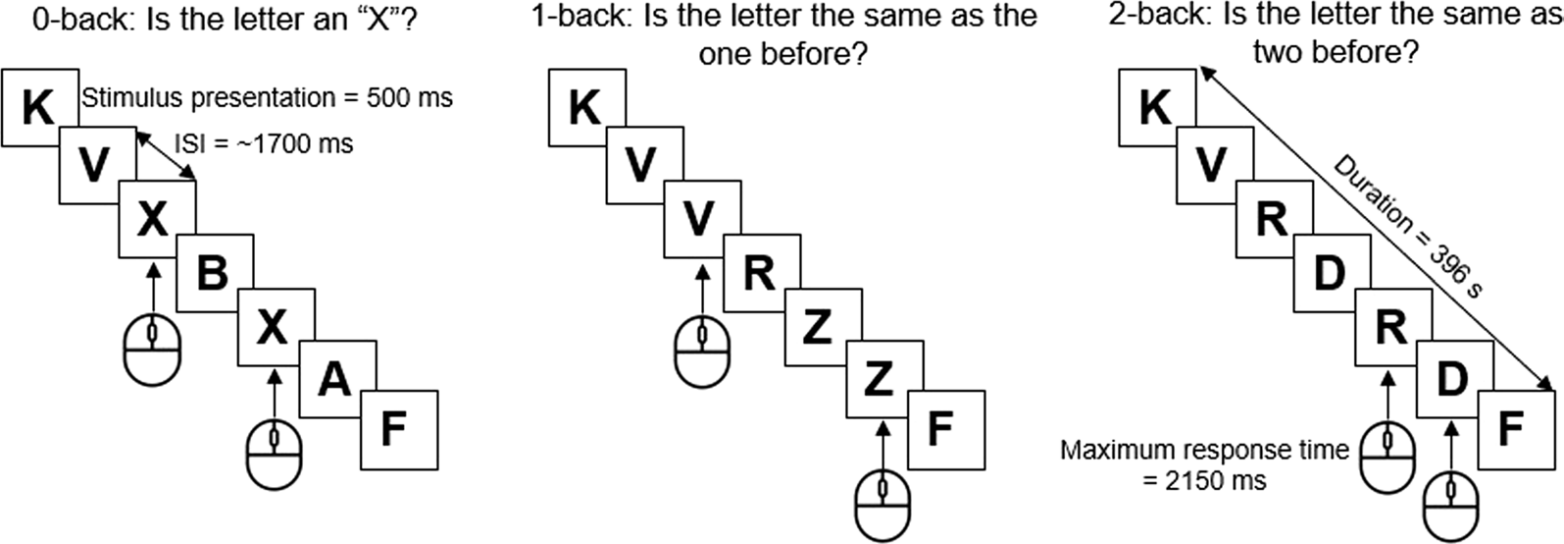
Design of the *n*-back test. ISI, interstimulus interval

In the *n*-back test, EEG was recorded while the participants were shown with a series of letters and instructed to press a button if the current stimulus was the same as the item presented *n* positions back (Fig. 1). The cognitive load increases with increased number, but the perceptual and motor demands remain the same. In this study, the 0-back, 1-back, and 2-back tests were administered (Fig. 1). The 0-back test was used as the control condition [27, 28]. The 1-back test requires the participant to passively store and update information in working memory. The 2-back test requires constant switching from the focus of attention to short-term memory [27]. Higher levels of difficulty require continuous mental effort to update information of new stimuli while maintaining representations of recently presented stimuli [29].

During the test, participants sat in a comfortable chair at 26 inches in front of a computer screen with the center of the screen at eye level. White letters appeared on the black screen. Participants completed a practice trial of 3 targets and 7 nontargets prior to each test. These practice sessions were repeated until the participants felt comfortable with the instructions. The actual test consisted of 60 trials that required a response by pressing the left mouse button (target, 33.3%) with their right index finger and 120 trials for which a response was not required (nontarget, 66.7%). Each letter was presented for 500 ms on the computer screen followed by a blank interstimulus interval for 1700 ms, with a random jitter of ± 50 ms. The allowed maximum response time was 2150 ms. The total task time was about 400 s. The number of correct responses (accuracy) and response times in the correct response trials were taken as the main behavioral outcome measures.

#### P3 ERP and event-related power

Continuous EEG was acquired using a Magstim EGI high-density system from 256 scalp electrodes, digitized at 1000 Hz. Data were online referenced to Cz and filtered using a 30 Hz low-pass filter and a 0.5 Hz high-pass filter in the EGI software. Although Kappenman and Luck recommend 0.10 Hz as the high-pass filter for EEG systems in P3 ERP studies [30], we used 0.5 Hz to account for the minimum high-pass filter threshold of 0.3 Hz set by the EGI system and to minimize the roll-off effect. All other EEG processing was done in EEGLab [31] and in ERPLab [32]. Recordings from electrodes around the face were first removed, leaving 183 electrode channels in the processing pipeline. Bad channels were removed through automatic identification and visual inspection of the EEG data. Various artifacts unrelated to cognitive functions, including ocular and muscular movement or cardiovascular signals, were identified and removed using independent component analysis. The stimulus-locked ERPs were extracted from the *n*-back tests and segmented into epochs of 100 ms before to 1000 ms after the stimulus onset, and baseline-corrected using the prestimulus interval. Epochs of incorrect and missed responses were removed from the analyses. Signals from bad electrodes were then interpolated using surrounding electrode data. Scalp locations and measurement windows for the P3 component were determined based on their spatial extent and latency after inspection of grand average waveforms. P3 peak amplitude of the task difference effect was considered the main outcome variable. The task difference effect was calculated by subtracting the average ERP elicited by targets from the average ERP elicited by nontargets (nontarget—target) for each participant. We also calculated P3 peak latency of the task difference effect as well as P3 peak amplitude and latency of the targets and nontargets. The P3-component time-window was established between 250 and 650 ms for all three tests. The average event-related power was identified in four frequency bands: delta [2–4 Hz], theta [4–8 Hz], alpha [8–12 Hz] and beta [12–30 Hz] [33]. Because of the involvement of prefrontal cortex in working memory, we analyzed P3 ERP from Fz, but also from Cz and Pz sites. Cz was interpolated using the surrounding five channels after re-referencing offline to the linked mastoids. No participants were excluded from analyses due to artifacts.

#### Data analysis

Descriptive analysis including mean (standard deviation), median (Q1–Q3), and frequency count of participants’ general demographics, performance measures, and ERP data was performed as appropriate. Unpaired *t*-tests, Median tests, and Chi-square tests were used to compare descriptive variables and performance in cognitive tests. We conducted linear mixed models to determine the effect of Aβ on P3 and event-related power at channel Fz. We used a random intercept term with a subject-specific coefficient to adjust for correlation between measures within subjects. Group (CNAβ+ and CNAβ−) and *n*-back difficulty (0, 1, 2) were entered as main effects. Interaction effects of group × *n*-back were also examined. Bonferroni correction was applied for pairwise comparisons. Residual analysis was used to validate model assumptions. Variables were transformed to their log function when residuals were not normally distributed. We entered age, sex, education, and MOCA as potential covariates in a separate linear mixed model. These analyses were repeated for channels Cz and Pz. In addition, linear mixed models were employed to investigate the main effects of group and condition (*n*-back) on the average event-related power in the delta, theta, alpha, and beta bands, and on performance in the *n*-back tests (response time and accuracy). Correlations of the mean Aβ standard uptake value ratio (SUVR) and the SUVR of six predefined regions (anterior cingulate, posterior cingulate, precuneus, inferior medial frontal, lateral temporal, and superior parietal cortex) with the P3 peak amplitude and latency of the task difference (nontarget—target) in each *n*-back test at channels Fz, Cz, and Pz were analyzed with Pearson *r* correlation coefficient. *P* < 0.05 was considered significant. Analyses were performed using SAS 9.4 and SAS Enterprise Guide 8.2 softwares.

## Results

### Participant characteristics

We recruited 17 CNAβ+ (age: 73 ± 5 years; 11 (65%) females; MOCA: 26 ± 2) and 17  CNAβ− (age: 75 ± 6; 12 (71%) females; MOCA: 28 ± 2) participants. MOCA scores ranged between 25 and 30. In the CNAβ+ group, two participants scored below 26 on the MOCA (22 and 23), and one participant was African American. All others identified their race as White. Two participants identified their ethnicity as Hispanic or Latino. No differences were observed for age, sex, and MOCA score between groups (Table 1).

**Table 1:** Comparison of descriptive, clinical, and performance variables between CNAβ+ and CNAβ− groups

Variable	CNAβ+ (*n* = 17)	CNAβ− (*n* = 17)	*P* value
Age	73 ± 5	75 ± 6	0.34^a^
Sex, females (%)	11 (65)	12 (71)	0.71^b^
MOCA	26 ± 2	28 ± 2	0.12^a^
0-back, response time (ms)	510 ± 114	510 ± 149	0.98^a^
0-back, accuracy (#)	60 (60–60)	60 (60–60)	0.29^c^
1-back, response time (ms)	543 ± 125	546 ± 95	0.96^a^
1-back, accuracy (#)	59 (57–59)	58 (57–60)	0.30^c^
2-back, response time (ms)	673 ± 143	665 ± 134	0.86^a^
2-back, accuracy (#)	52 (49–54)	50 (39–55)	0.31^c^

CNAβ+, cognitively normal, beta-amyloid positive; CNAβ−, cognitively normal, beta-amyloid negative; MOCA, Montreal Cognitive Assessment. Variables are described as mean ± standard deviation; median (Q1–Q3), or number (frequency). ^a^Independent *t*-test; ^b^Chi-square test; ^c^Median test. ^#^Number of correct response

We first analyzed differences in the accuracy and response time in the *n*-back test (Table 1). The linear mixed models showed no main group effects on response time (*P* = 0.36) and accuracy (*P* = 0.91).

### P3 grand average waveforms

**Fig. 2:**
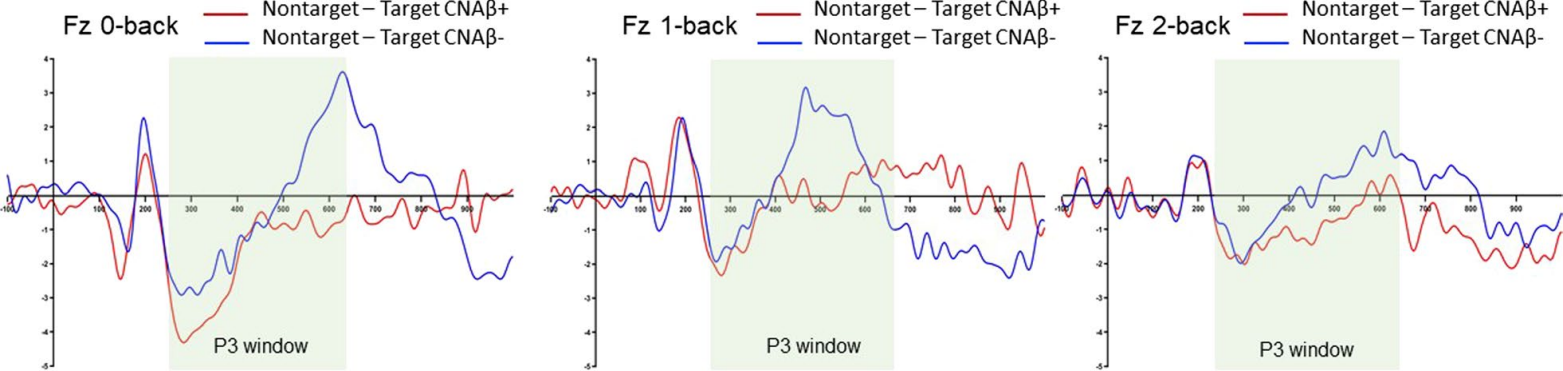
Grand average waveforms of the target difference effect (nontarget−target) for the three *n*-back tests between CNAβ+ (top) and CNAβ− (bottom) at channel Fz

The grand average peak P3 amplitudes of the task difference effect (nontarget—target) of the two groups for each *n*-back condition at channels Fz, Cz, and Pz are shown in Additional file 1: Table S1. Figure 2 shows that the task difference effect of the peak amplitude at channel Fz was lower in CNAβ+ compared to CNAβ− (*P* = 0.048, *P* = 0.05 after adjusting for age, sex, and MoCA scores). No other effects were found for peak amplitude. Additional file 1: Table S2 shows that the P3 latency of the task difference effect at channel Fz was sensitive to changes in cognitive demand (non-adjusted *P* = 0.047; adjusted *P* = 0.05).

**Fig. 3:**
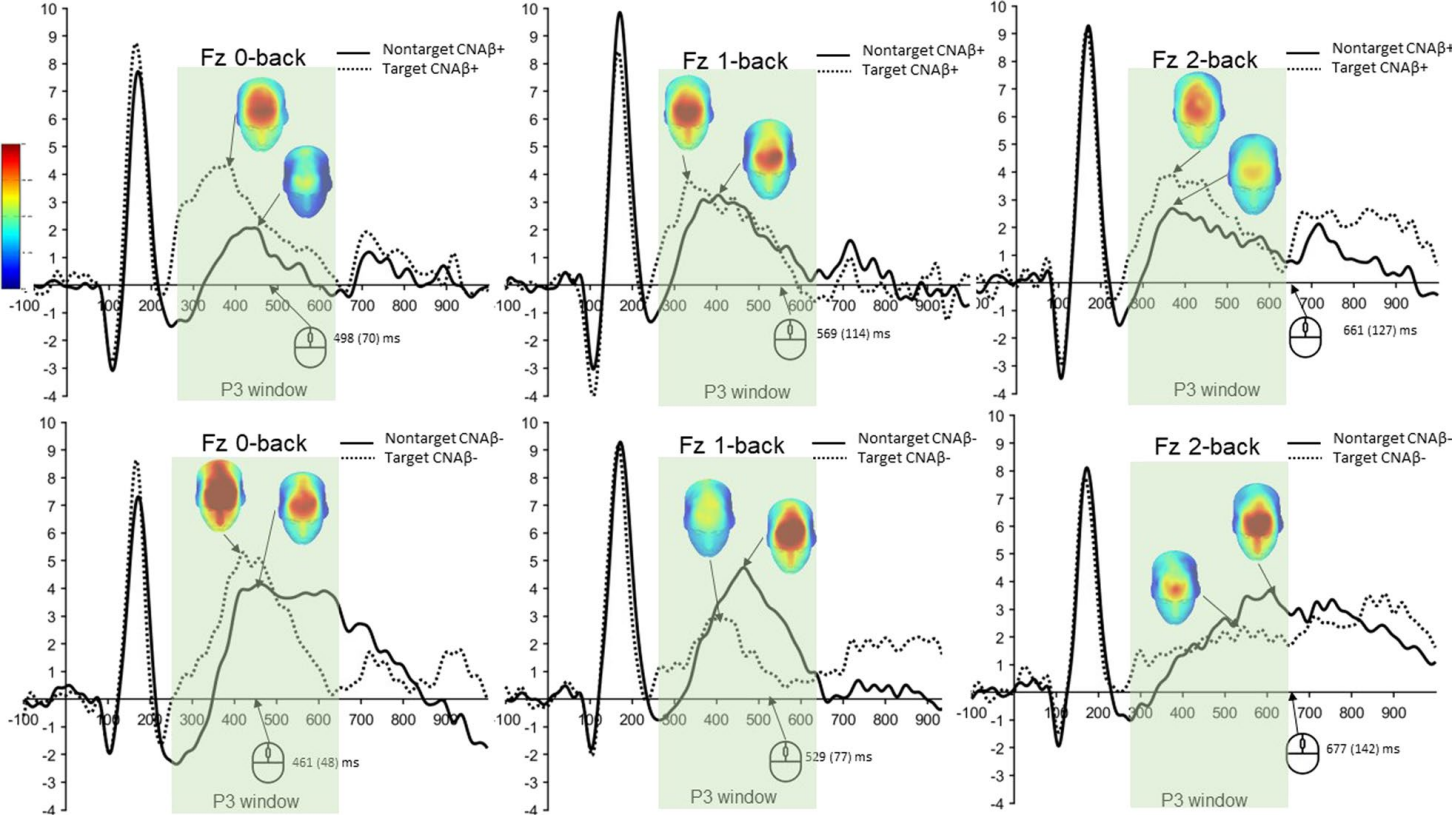
Grand average waveforms with P3 peak scalp maps of the target and nontarget responses for the three *n*-back tests between CNAβ+ (top) and CNAβ− (bottom) at channel Fz. Behavioral response times are indicated by the computer mouse. The 3D scalp maps are facing down and most of the P3 effects (red color) are distributed in the frontal area

The grand average waveforms of the targets and nontargets at channel Fz of both groups are depicted in Fig. 3. Linear mixed model analysis revealed shorter P3 latency for nontargets (non-adjusted *P* = 0.006; adjusted *P* = 0.006) at channel Fz in CNAβ+ (Additional file 1: Table S2).

No other effects were found at channels Cz and Pz, except for the peak latency of the nontargets at channel Cz that produced significant group effects (non-adjusted *P* = 0.04; adjusted *P* = 0.04).

## P3 event-related power

Power in each of the frequency bands for each of the three *n*-back conditions at channels Fz, Cz, and Pz is detailed in Additional file 1: Table S3.

At channel Fz, the CNAβ+ participants exhibited lower power in the delta band for nontargets (unadjusted *P* = 0.04; adjusted *P* = 0.08, with age [*P* = 0.01] and MOCA scores [*P* = 0.007] contributing significantly to the model), compared to the CNAβ− participants.

At channel Cz, the CNAβ+ participants exhibited higher power in the theta band for the task difference effect (unadjusted *P* = 0.05; adjusted *P* = 0.09). In addition, higher power was observed in the alpha band for nontargets (unadjusted *P* = 0.05; adjusted *P* = 0.03), targets (unadjusted *P* = 0.04; adjusted *P* = 0.03), and the task difference effect (unadjusted *P* = 0.03; adjusted *P* = 0.09, with age [ *P* = 0.02] contributing significantly to the model).

At channel Pz, the CNAβ+ participants exhibited higher power in the theta band for nontargets (unadjusted *P* = 0.05; adjusted *P* = 0.07) and task difference effect (unadjusted *P* = 0.03; adjusted *P* = 0.11). Likewise, higher power in the alpha band was observed in the CNAβ+ group (unadjusted* P* = 0.03; adjusted *P* = 0.03).

Analyses of the beta band did not show significant effects.

### Correlation between amyloid and P3 ERP

**Fig. 4:**
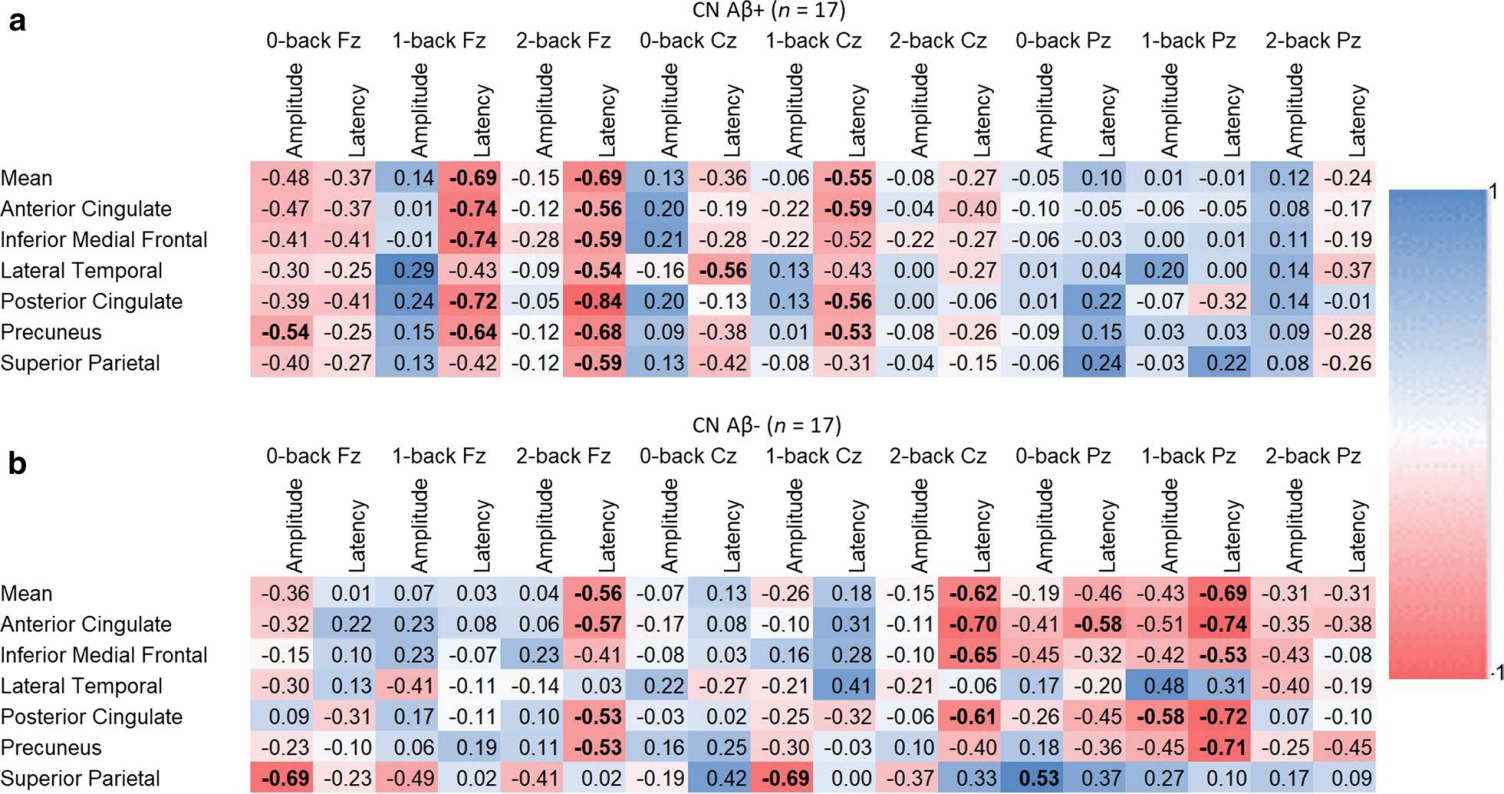
Pearson *r* correlations between beta-amyloid (Aβ) standard uptake value ratio and P3 event-related potential latency and amplitude for each *n*-back test at channel Fz, Cz, and Pz. The color heat map shows the magnitude of the correlations in the positive (green) and negative (red) direction. Bolded values are significant (*P* < 0.05). Top panel **a** shows correlations for the cognitively normal, elevated (CNAβ+) group; bottom panel **b** shows the correlations for the cognitively normal, non-elevated (CNAβ−) group

The correlation table shows stronger correlation of SUVR with ERP latency than with amplitude of the task difference effect (Fig. 4). Absolute Pearson *r* correlation coefficient of 0.53 and higher indicates significant correlation (*P* < 0.05). The mean Aβ SUVR of the CNAβ+ group correlated negatively with the P3 peak latency in the 1-back (*r* =  − 0.69; *P* = 0.003) and 2-back (*r* =  − 0.69; *P* = 0.004) tests at Fz. Aβ SUVR in the anterior cingulate cortex (*r* =  − 0.74; *P* = 0.009), inferior medial frontal lobe (*r* =  − 0.74; *P* = 0.001), posterior cingulate cortex (*r* =  − 0.72; *P* = 0.002) and precuneus (*r* =  − 0.64; *P* = 0.005) correlated strongly with the P3 peak latency in the 1-back test at channel Fz. Similarly, strong correlations were observed between Aβ SUVR in the posterior cingulate cortex (− 0.84; *P* = 0.0001), precuneus (*r* =  − 0.68; *P* = 0.005), inferior medial frontal lobe (*r* = -0.59; *P* = 0.02), superior parietal lobe (*r* =  − 0.59; *P* = 0.02), anterior cingulate cortex (*r* =  − 0.56; *P* = 0.03), and lateral temporal lobe (*r* =  − 0.54; *P* = 0.04) and the P3 peak latency in the 2-back test at channel Fz. Overall, the magnitude of correlations between Aβ SUVR and P3 peak amplitude and latency was smaller at channels Cz and Pz than at Fz in CNAβ+.

Aβ SUVR also correlated with the P3 peak amplitude of several *n*-back conditions in the CNAβ− group (Fig. 3b). SUVR in the superior parietal cortex correlated negatively with P3 peak amplitude of the 0-back test at channel Fz (*r* =  − 0.69; *P* = 0.007) and of the 1-back test at channel Cz (*r* =  − 0.69; *P* = 0.006). SUVR of the posterior cingulate cortex correlated with P3 peak amplitude of the 1-back test at Pz (*r* =  − 0.58; *P* = 0.03). The mean SUVR correlated with P3 peak latency of the 2-back test at Fz (*r* =  − 0.56; *P* = 0.04) and Cz (*r =* − 0.62; *P* = 0.01) and of the 1-back test at Pz (*r* =  − 0.69; *P* = 0.006). Similar magnitudes of correlation were observed for subregions anterior cingulate cortex, inferior medial frontal lobe, posterior cingulate cortex, and precuneus.

## Discussion

The goal of this study was to compare neuronal excitability during working memory of incremental cognitive demand between CNAβ+ and CNAβ− older adults. We demonstrated differences in the P3 ERP (decreased peak P3 ERP of the task difference) as well as changes in the event-related power (lower power in the low-frequency bands [delta] and higher power in the mid-range-frequency bands [theta, alpha]) in CNAβ+ adults, compared with  CNAβ−. Cognitive load was not associated with the differences in P3 ERP amplitude between the two groups. In addition, we found strong correlations between Aβ deposits in cortical brain regions and P3 ERP. These findings point towards evidence of hyperexcitability in CNAβ+. However, this hyperexcitability did not appear to affect behavioral performance as no differences were found in accuracy and response times on the *n*-back test.

Our study demonstrated lower delta event-related power in the frontal midline channel, along with an increase in theta and alpha event-related power in the central and parietal midline channels in CNAβ+. These results confirm the preclinical AD animal model studies showing that hyperexcitability is related to early breakdown of low-frequency waves [4]. The increased event-related power in alpha and theta frequencies in CNAβ+ contrasts the changes in event-related power found in older adults with cognitive impairments. While an increase has been found in absolute theta power [34–36], the event-related theta power was significantly lower in response to cognitive load in MCI and AD compared to controls, reflecting hypoexcitability [36]. The combined lower event-related delta power and higher event-related alpha and theta power suggest that CNAβ+ older adults may exhibit neuronal hyperexcitability. A previous study investigating resting-state spectral power has classified CN older adults with subjective memory complaints according to their Aβ burden (+ or −) and associated neurodegeneration (+ or −) into four respective categories, and found a U-shaped distribution in delta power and an inverse U-shaped distribution in gamma power, most pronounced in CN individuals with signs of neurodegeneration [11]. In addition, the presence of neurodegeneration is associated with a decrease in lower-frequency waves (delta) and an increase in higher-frequency waves (beta and gamma) in the fronto-central regions [11]. Yet, there are no associations between Aβ load and spectral power in the absence of neurodegeneration.

The similarities in spectral power in the different frequency bands between the previous study [11] and ours imply that our group of CNAβ+ participants may have shown early signs of neurodegeneration. However, we cannot confirm this assumption as we did not formally assess neurodegeneration. Another potential explanation is that changes in power may appear earlier in the disease process (i.e., in Aβ+ with no neurodegeneration) under cognitive load as opposed to the resting state. Although no interaction effects of group by *n*-back were found, visual inspection of the P3 waveforms showed that the differences between CNAβ+ and CNAβ− were most obvious under highest cognitive load. However, our study may have been underpowered to elicit these differences statistically.

Although older adults with elevated amyloid may exhibit neuronal hyperexcitability, these compensatory processes do not result in more efficient neural processes. Our results showed that the CNAβ+ group exhibited a smaller P3 amplitude of the task difference effect, suggesting less efficient stimulus processing compared to  CNAβ−. The absence of a clear effect of task difficulty on P3 amplitude and latency in the 2-back test in elevated amyloid also implies a lack of appropriate reallocation of cognitive resources away from stimulus evaluation. These findings, along with the non-significant differences in behavioral outcomes, suggest that the hyperexcitability is a non-functional compensation on neural level due to increased Aβ deposition, and may result in less efficient cognitive processing of working memory.

Our results suggest a direct link between average and regional Aβ burden and electrophysiological activity, particularly in the frontal cortex in CNAβ+. This is consistent with animal studies that found hyperactive neurons exclusively around the Aβ plaques [5], suggesting that Aβ exerts toxic effects on surrounding neurons and synapses, thereby disturbing their function and perhaps leading to dementia [37]. In particular, soluble Aβ oligomers have been shown to affect neuronal excitability in animal models and in vitro in humans [38]. However, no causal inferences can be made from our results. Longitudinal studies are required to identify the effect of Aβ burden on the relative postsynaptic excitation, and the role of P3 as a biomarker of pathophysiological, clinical, and functional decline. Future studies should investigate whether a relative increase in excitatory neurotransmitters, particularly glutamate, drives the link between Aβ burden and P3 ERP in preclinical AD. If confirmed, EEG metrics may be used as endpoints for mechanistic studies evaluating hypotheses related to autophagy [39], mitophagy [40], and selective neuronal vulnerability of AD [41], and for translational intervention studies aiming to reduce Aβ burden with pharmacological treatment [42], behavioral interventions (e.g., exercise), etc. [43]

Limitations of this study include the relatively small sample size and the long interval between PET scan and EEG testing. We cannot rule out the possibility that some CNAβ− participants might have converted to CNAβ+. The projected conversion rate from Aβ− to Aβ+ is about 4% per year [44], showing stability of cortical Aβ in the vast majority of older adults. However, we plan to conduct a future study where the PET scan and EEG assessment are conducted close in time. In addition, two participants in the CNAβ+ group scored below 26 on MoCA, which may indicate a change in cognitive status since their comprehensive cognitive assessment. Therefore, our results should be interpreted with caution. We also corrected for multiplicity by design. To account for the multiplicity, we designated in advance of the study a single linear mixed model with the task difference effect of P3 ERP as our primary result. All other tests were designated as secondary and presented in full to provide complete transparency. However, we did implement standard multiplicity adjustments within our linear mixed models. We chose the *n*-back test to test our hypotheses as working memory is regarded a core cognitive function sensitive to aging and early neurodegeneration, upon which higher-order cognitive skills, such as attention, decision making, and planning are built [45]. Our results are therefore unique to working memory and cannot be generalized to other domains of cognitive functions that are relevant to AD.

## Conclusion

Older adults with normal cognition and elevated Aβ show neuronal hyperexcitability under cognitive load. This hyperexcitability affects cognitive processes indexed by the P3 ERP. Future studies are required to elucidate the causal effects between Aβ depositions and neural excitability.

## Supplementary Information

**Additional file 1**: **Table S1**. P3 peak amplitude and latency at Fz/Cz/Pz across testing conditions of CNAβ+ and  CNAβ−. **Table S2**. Event-related power (µV/Hz2) at Fz/Cz/Pz, across testing conditions of CNAβ+ and  CNAβ− (Mean ± SD). **Table S3**. Event-related power (μV/Hz2) of CNAβ+ (*n* = 17) and CNAβ− (*n* = 17) at Fz/Cz/Pz, across testing conditions (Mean ± SD).

## Data Availability

The datasets used and/or analyzed during the current study are available from the corresponding author on reasonable request.

## Abbreviations

Aβ: Beta-amyloid
AD: Alzheimer’s disease
CN: Cognitively normal
EEG: Electro-encephalography
MCI: Mild cognitive impairment
MoCA: Montreal Cognitive Assessment
ERP: Event-related potential
ISI: Interstimulus interval
PET: Positron emission tomography
SUVR: Standard uptake value ratio
