# Supplementary material for: EEG/ERP evidence of possible hyperexcitability in older adults with elevated beta-amyloid
Source: Transl Neurodegener. 2022 Feb 9;11:8. doi: 10.1186/s40035-022-00282-5 (PMC8827181; doi:10.1186/s40035-022-00282-5)
Supplement: Supplementary file 1 — Additional file 1: Table S1. P3 peak amplitude and latency at Fz/Cz/Pz across testing conditions of CNAβ+ and CNAβ−. Table S2. Event-related power (µV/Hz2) at Fz/Cz/Pz, across testing conditions of CNAβ+ and CNAβ− (Mean ± SD). Table S3. Event-related power (μV/Hz2) of CNAβ+ (n = 17) and CNAβ− (n = 17) at Fz/Cz/Pz, across testing conditions (Mean ± SD). [file 40035_2022_282_MOESM1_ESM.pdf]

| Supplementary Table 1. Mean ± standard deviation of the grand average P3 peak amplitude of CNAβ+ and CNAβ- at Fz/Cz/Pz across testing conditions | | | |
|---|---|---|---|
| Variables | | CNAβ+ (n= 17)<br>Mean ± Std. Dev. | CNAβ- (n = 17)<br>Mean ± Std. Dev. |
| Fz Amplitude (µV) | | | |
| 0-back | Nontarget | 2.068 ± 3.312 | 4.156 ± 3.124 |
| | Target | 4.374 ± 3.701 | 5.302 ± 3.825 |
| | Difference | -0.437 ± 2.447 | 3.616 ± 2.471 |
| 1-back | Nontarget | 3.248 ± 4.574 | 4.748 ± 8.184 |
| | Target | 3.758 ± 4.620 | 2.962 ± 4.370 |
| | Difference | 1.043 ± 2.187 | 3.170 ± 6.262 |
| 2-back | Nontarget | 2.686 ± 2.211 | 3.669 ± 6.709 |
| | Target | 3.914 ± 3.309 | 2.358 ± 2.694 |
| | Difference | 0.537 ± 1.592 | 1.851 ± 5.785 |
| Cz Amplitude (µV) | | | |
| 0-back | Nontarget | 2.424 ± 1.990 | 4.416 ± 7.628 |
| | Target | 2.325 ± 2.980 | 3.018 ± 1.452 |
| | Difference | 0.484 ± 2.931 | 1.887 ± 6.130 |
| 1-back | Nontarget | 2.794 ± 2.187 | 2.978 ± 2.126 |
| | Target | 2.405 ± 3.056 | 2.264 ± 2.208 |
| | Difference | 1.078 ± 2.931 | 1.887 ± 5.785 |
| 2-back | Nontarget | 1.762 ± 1.508 | 1.926 ± 6.730 |
| | Target | 2.475 ± 2.574 | 1.821 ± 4.806 |
| | Difference | 0.314 ± 1.464 | 0.726 ± 2.282 |
| Pz Amplitude (µV) | | | |
| 0-back | Nontarget | 1.246 1.709 | 1.364 ± 1.362 |
| | Target | 2.646 ± 1.766 | 3.493 ± 3.123 |
| | Difference | 0.333 ± 2.266 | 0.589 ± 2.100 |
| 1-back | Nontarget | 2.035 ± 2.657 | 1.958 ± 1.571 |
| | Target | 2.410 ± 2.540 | 3.068 ± 2.747 |
| | Difference | 0.499 ± 1.909 | 0.409 ± 1.401 |
| 2-back | Nontarget | 1.093 ± 1.9656 | 2.144 ± 2.281 |
| | Target | 1.021 ± 2.130 | 2.226 ± 2.121 |
| | Difference | 0.824 ± 1.621 | 0.733 ± 1.178 |
| Abbreviations: CNAβ, cognitively normal, beta-amyloid | | | |

| Supplementary Table 2. Peak P3 latency at Fz/Cz/Pz across testing conditions of CNAβ+ and CNAβ- | | | |
|---|---|---|---|
| Variables | | CNAβ+<br>Mean ± Std. Dev. | CNAβ-<br>Mean ± Std. Dev. |
| Fz Latency (ms) | | | |
| 0-back | Nontarget | 418 ± 82 | 461 ± 80 |
| | Target | 399 ± 91 | 405 ± 98 |
| | Difference | 497 ± 83 | 470 ± 84 |
| 1-back | Nontarget | 415 ± 81 | 461 ± 80 |
| | Target | 401 ± 90 | 403 ± 100 |
| | Difference | 491 ± 85 | 469 ± 84 |
| 2-back | Nontarget | 418 ± 79 | 463 ± 81 |
| | Target | 404 ± 92 | 403 ± 100 |
| | Difference | 490 ± 86 | 476 ± 78 |
| Cz Latency (ms) | | | |
| 0-back | Nontarget | 420 ± 86 | 455 ± 89 |
| | Target | 409 ± 86 | 431 ± 89 |
| | Difference | 476 ± 99 | 438 ± 104 |
| 1-back | Nontarget | 416 ± 85 | 452 ± 93 |
| | Target | 413 ± 86 | 429 ± 89 |
| | Difference | 472 ± 98 | 443 ± 103 |
| 2-back | Nontarget | 418 ± 84 | 458 ± 90 |
| | Target | 417 ± 90 | 426 ± 92 |
| | Difference | 471 ± 98 | 450 ± 101 |
| Pz Latency (ms) | | | |
| 0-back | Nontarget | 380 ± 108 | 404 ± 114 |
| | Target | 408 ± 82 | 404 ± 93 |
| | Difference | 421 ± 122 | 387 ± 112 |
| 1-back | Nontarget | 379 ± 105 | 404 ± 113 |
| | Target | 406 ± 83 | 403 ± 93 |
| | Difference | 421 ± 122 | 391 ± 115 |
| 2-back | Nontarget | 377 ± 106 | 411 ± 115 |
| | Target | 410 ± 84 | 405 ± 95 |
| | Difference | 415 ± 121 | 400 ± 118 |
| Abbreviations: CNAβ, cognitively normal, beta-amyloid | | | |

Supplementary Table 3. Event-related power (μV/Hz$^2$) of CNAβ+ (n = 17) and CNAβ- (n = 17) at Fz/Cz/Pz, across testing conditions. (Mean ± Std. Dev.)

| | | Fz Channel | | Cz Channel | | Pz Channel | |
|---|---|---|---|---|---|---|---|
| | | CNAβ+ | CNAβ- | CNAβ+ | CNAβ- | CNAβ+ | CNAβ- |
| Delta Band | | | | | | | |
| 0-back | Nontarget | 0.443 ± 0.455 | 1.916 ± 3.681 | 0.328 ± 0.322 | 0.601 ± 1.111 | 0.086 ± 0.132 | 0.096 ± 0.153 |
| | Target | 0.371 ± 0.328 | 3.763 ± 3.979 | 0.285 ± 0.305 | 1.519 ± 5.064 | 0.207 ± 0.289 | 0.319 ± 0.799 |
| | Difference | 0.397 ± 0.390 | -1.288 ± 1.392 | 0.129 ± 0.131 | -0.800 ± 4.018 | 0.128 ± 0.215 | -0.043 ± 0.709 |
| 1-back | Nontarget | 0.600 ± 0.787 | 1.343 ± 2.841 | 0.289 ± 0.352 | 0.273 ± 0.217 | 0.078 ± 0.081 | 0.069 ± 0.086 |
| | Target | 0.593 ± 1.206 | 0.359 ± 0.464 | 0.167 ± 0.158 | 0.199 ± 0.189 | 0.140 ± 0.174 | 0.142 ± 0.237 |
| | Difference | 0.526 ± 0.937 | 1.455 ± 3.763 | 0.150 ± 0.224 | 0.116 ± 0.149 | 0.124 ± 0.164 | 0.073 ± 0.100 |
| 2-back | Nontarget | 0.309 ± 0.286 | 2.217 ± 6.668 | 0.213 ± 0.308 | 0.364 ± 0.718 | 0.059 ± 0.091 | 0.087 ± 0.105 |
| | Target | 0.413 ± 0.580 | 0.797 ± 2.353 | 0.245 ± 0.449 | 0.223 ± 0.381 | 0.100 ± 0.176 | 0.059 ± 0.064 |
| | Difference | 0.215 ± 0.314 | 1.518 ± 4.346 | 0.072 ± 0.082 | 0.135 ± 0.334 | 0.046 ± 0.073 | 0.034 ± 0.059 |
| Theta Band | | | | | | | |
| 0-back | Nontarget | 0.168 ± 0.145 | 0.208 ± 0.202 | 0.117 ± 0.167 | 0.102 ± 0.095 | 0.039 ± 0.081 | 0.022 ± 0.026 |
| | Target | 0.145 ± 0.096 | 0.278 ± 0.539 | 0.107 ± 0.133 | 0.126 ± 0.206 | 0.051 ± 0.088 | 0.033 ± 0.046 |
| | Difference | 0.085 ± 0.073 | -0.006 ± 0.368 | 0.050 ± 0.064 | 0.001 ± 0.130 | 0.035 ± 0.048 | 0.007 ± 0.027 |
| 1-back | Nontarget | 0.236 ± 0.230 | 0.167 ± 0.096 | 0.150 ± 0.204 | 0.085 ± 0.052 | 0.049 ± 0.089 | 0.023 ± 0.026 |
| | Target | 0.182 ± 0.136 | 0.151 ± 0.155 | 0.128 ± 0.167 | 0.079 ± 0.048 | 0.051 ± 0.089 | 0.030 ± 0.038 |
| | Difference | 0.125 ± 0.264 | 0.081 ± 0.122 | 0.040 ± 0.057 | 0.027 ± 0.019 | 0.027 ± 0.048 | 0.018 ± 0.024 |
| 2-back | Nontarget | 0.203 ± 0.193 | 0.222 ± 0.391 | 0.148 ± 0.196 | 0.072 ± 0.059 | 0.055 ± 0.114 | 0.016 ± 0.018 |
| | Target | 0.185 ± 0.166 | 0.189 ± 0.351 | 0.140 ± 0.179 | 0.062 ± 0.052 | 0.054 ± 0.104 | 0.020 ± 0.023 |
| | Difference | 0.050 ± 0.069 | 0.052 ± 0.063 | 0.035 ± 0.062 | 0.015 ± 0.012 | 0.017 ± 0.021 | 0.009 ± 0.011 |

| | | | | | | | |
|---|---|---|---|---|---|---|---|
| Alpha Band | | | | | | | |
| 0-back | Nontarget | 0.050 ± 0.041 | 0.052 ± 0.054 | 0.034 ± 0.037 | 0.029 ± 0.031 | 0.018 ± 0.032 | 0.008 ± 0.009 |
| | Target | 0.085 ± 0.070 | 0.083 ± 0.089 | 0.061 ± 0.075 | 0.046 ± 0.047 | 0.025 ± 0.038 | 0.013 ± 0.017 |
| | Difference | 0.039 ± 0.034 | 0.017 ± 0.053 | 0.031 ± 0.037 | 0.011 ± 0.030 | 0.018 ± 0.031 | 0.009 ± 0.020 |
| 1-back | Nontarget | 0.059 ± 0.045 | 0.046 ± 0.036 | 0.045 ± 0.052 | 0.026 ± 0.019 | 0.016 ± 0.024 | 0.008 ± 0.008 |
| | Target | 0.075 ± 0.070 | 0.060 ± 0.051 | 0.056 ± 0.076 | 0.035 ± 0.024 | 0.024 ± 0.045 | 0.012 ± 0.012 |
| | Difference | 0.028 ± 0.022 | 0.030 ± 0.033 | 0.019 ± 0.024 | 0.016 ± 0.017 | 0.010 ± 0.015 | 0.007 ± 0.005 |
| 2-back | Nontarget | 0.055 ± 0.056 | 0.039 ± 0.024 | 0.045 ± 0.061 | 0.022 ± 0.016 | 0.019 ± 0.036 | 0.006 ± 0.007 |
| | Target | 0.084 ± 0.091 | 0.048 ± 0.034 | 0.065 ± 0.084 | 0.026 ± 0.018 | 0.023 ± 0.037 | 0.013 ± 0.017 |
| | Difference | 0.034 ± 0.049 | 0.015 ± 0.022 | 0.019 ± 0.028 | 0.008 ± 0.009 | 0.008 ± 0.010 | 0.009 ± 0.016 |
| Beta Band | | | | | | | |
| 0-back | Nontarget | 0.006 ± 0.007 | 0.007 ± 0.007 | 0.004 ± 0.006 | 0.003 ± 0.003 | 0.002 ± 0.002 | 0.001 ± 0.001 |
| | Target | 0.009 ± 0.007 | 0.014 ± 0.015 | 0.005 ± 0.006 | 0.006 ± 0.006 | 0.002 ± 0.002 | 0.003 ± 0.003 |
| | Difference | 0.009 ± 0.007 | 0.010 ± 0.015 | 0.006 ± 0.006 | 0.004 ± 0.006 | 0.003 ± 0.002 | 0.003 ± 0.005 |
| 1-back | Nontarget | 0.006 ± 0.004 | 0.005 ± 0.003 | 0.004 ± 0.004 | 0.003 ± 0.002 | 0.002 ± 0.003 | 0.002 ± 0.001 |
| | Target | 0.009 ± 0.008 | 0.008 ± 0.005 | 0.005 ± 0.005 | 0.004 ± 0.003 | 0.003 ± 0.004 | 0.002 ± 0.002 |
| | Difference | 0.009 ± 0.009 | 0.009 ± 0.007 | 0.006 ± 0.007 | 0.005 ± 0.004 | 0.003 ± 0.003 | 0.003 ± 0.002 |
| 2-back | Nontarget | 0.006 ± 0.004 | 0.005 ± 0.003 | 0.004 ± 0.004 | 0.003 ± 0.002 | 0.002 ± 0.002 | 0.002 ± 0.001 |
| | Target | 0.012 ± 0.016 | 0.008 ± 0.006 | 0.007 ± 0.012 | 0.004 ± 0.002 | 0.004 ± 0.005 | 0.003 ± 0.003 |
| | Difference | 0.013 ± 0.019 | 0.007 ± 0.008 | 0.008 ± 0.016 | 0.004 ± 0.004 | 0.004 ± 0.005 | 0.003 ± 0.004 |

Abbreviations: CNAβ, cognitively normal beta-amyloid
